# Development of a forecasting model for brucellosis spreading in the Italian cattle trade network aimed to prioritise the field interventions

**DOI:** 10.1371/journal.pone.0177313

**Published:** 2017-06-27

**Authors:** L. Savini, L. Candeloro, A. Conte, F. De Massis, A. Giovannini

**Affiliations:** Istituto Zooprofilattico Sperimentale dell’Abruzzo e Molise, National and OIE Reference Laboratory for Brucellosis, OIE Collaborating Centre for Veterinary Training, Epidemiology, Food Safety and Animal Welfare, Via Campo Boario, Teramo, Italy; Universidad Rey Juan Carlos, SPAIN

## Abstract

Brucellosis caused by Brucella abortus is an important zoonosis that constitutes a serious hazard to public health. Prevention of human brucellosis depends on the control of the disease in animals. Livestock movement data represent a valuable source of information to understand the pattern of contacts between holdings, which may determine the inter-herds and intra-herd spread of the disease. The manuscript addresses the use of computational epidemic models rooted in the knowledge of cattle trade network to assess the probabilities of brucellosis spread and to design control strategies. Three different spread network-based models were proposed: the DFC (Disease Flow Centrality) model based only on temporal cattle network structure and unrelated to the epidemiological disease parameters; a deterministic SIR (Susceptible-Infectious-Recovered) model; a stochastic SEIR (Susceptible-Exposed-Infectious-Recovered) model in which epidemiological and demographic within-farm aspects were also modelled. Containment strategies based on farms centrality in the cattle network were tested and discussed. All three models started from the identification of the entire sub-network originated from an infected farm, up to the fifth order of contacts. Their performances were based on data collected in Sicily in the framework of the national eradication plan of brucellosis in 2009. Results show that the proposed methods improves the efficacy and efficiency of the tracing activities in comparison to the procedure currently adopted by the veterinary services in the brucellosis control, in Italy. An overall assessment shows that the SIR model is the most suitable for the practical needs of the veterinary services, being the one with the highest sensitivity and the shortest computation time.

## Introduction

Brucellosis is an important zoonotic disease caused by infection with bacteria of the Genus Brucella. Brucellosis in humans and animals is a worldwide problem and one of the most important zoonoses in the Mediterranean and Middle East regions. Although continuous progress has been achieved in its control, brucellosis still remains a major public health threat as well as a disease of great economic importance, and its control and eradication still remains a goal for Veterinary and Health Authorities in several countries [1]. Losses from the disease in food production include restrictions in commerce, slaughter and replacement of seropositive animals, as well as vaccination costs in countries in which this latter control method is applied. On the other hand, it is difficult to measure economic losses due to the disease in humans such as medical costs and reduced productivity [2]. The disease can be transmitted to humans directly by contact with infected animals or indirectly by contaminated dairy products. Prevention of human brucellosis depends primarily on control of the disease in animals [3].

In Italy, while central and northern provinces are declared officially brucellosis free, with limited numbers of northern cases reported annually [4,5], bovine brucellosis is endemic in the southern part of the country [6].

Susceptibility of cattle to infection is influenced by the age, sex and reproductive status of the individual animal. Susceptibility increases as stage of gestation increases [7,8]. The spread of the disease from one herd to another (inter-herd spread), and from one area to another, is primarily due to animal contacts at pastures or through animal movements between herds [6].

The control of brucellosis is based on the identification through active and passive surveillance of the infected herds. As soon as an infected herd has been detected, all animal movements from and to this herd are blocked and the infected animals within the herd are culled. Meanwhile, all herds that had contacts (through animal movements, pasture contacts, indirect contacts through exchange of materials or personnel, etc.) are identified and checked for the presence of infection. The tracing and checking of herds in-contact is a cumbersome activity that may require long time to be carried out. During this time the infection can further spread. Our objective was to study possible ways to prioritise the herds in-contact to limit the possible spread of the disease during this phase.

Therefore, modelling the spread of brucellosis requires knowledge about where and when animals move into and out respective herds, to be able to trace potentially infected animals.

Network analysis (NA), mathematical models, and in particular network models of disease spread, are promising tools for control-policy design, given that they can provide comprehensive quantitative representations of disease transmission pathways.

In the past, a wide range of analyses has been carried out with a particular focus on understanding the role played by the network's topological structure in the disease spread dynamics [9–11]

Network analysis provides tools for studying potential epidemic spread and identifying central and/or highly connected holdings (nodes) in a network of animal movements (arcs). In this way, the holdings that have a crucial role in the network could be targeted for surveillance activities in order to confine highly contagious diseases to restricted areas and preventing spread [12–14].

The centrality measures can be useful for planning and defining prevention activities but they are of limited or no value in case of control strategy of a disease already spread, such as that observed in our scenario of brucellosis epidemic.

Studies have demonstrated that mathematical modelling can aid the veterinary services in developing brucellosis control strategies.

The majority of these mathematical models are based on a compartmental model in which the individuals are grouped according to their disease status and some of them are embedded in animal movement networks [15,16–18]. However the influence of the topological structure of the system as well as many other complex aspects of the progression of the disease are neglected in these approaches.

The manuscript addresses the use of computational epidemic models rooted in the knowledge of cattle trade network to assess the probabilities of brucellosis spread and to design control strategies.

First, we analyse the impact of containment strategies based on farms centrality in the cattle network and then the network data is used to build three different spread network-based models: the DFC (Disease Flow Centrality) model based only on temporal cattle network structure and unrelated to the epidemiological disease parameters; a deterministic SIR (Susceptible-Infectious-Recovered) model; a stochastic SEIR (Susceptible-Exposed-Infectious-Recovered) model in which epidemiological and demographic within-farm aspects are also modelled.

The performance of these models was based on data collected in Sicily in the framework of the national eradication program of brucellosis in 2009.

## Material & methods

The analysis was carried out in two steps:

1. Construction of the transmission network (TN) starting from the seeding site (the first brucellosis outbreak, detected in Sicily during 2009, was used as origin node of the real network of animal movements) and subsequent network analysis to investigate the structure (or topology) and to calculate the main centrality measures of TN.
2. Implementation of three epidemiological models based on the real network of animal movements, namely: DFC (Disease Flow Centralities), deterministic SIR (Susceptible-Infectious-Recoverd) and a stochastic SEIR (Susceptible-Exposed-Infectious-Recoverd). To evaluate the performance of these models, field data of a brucellosis transmission chain occurred in Sicily in 2009 were used.

### Data source of Italian cattle movements and animal health information

Data on cattle populations and movements were collected from the Italian National Database for Animal Identification and Registration (NDB) which is managed by the Istituto Zooprofilattico Sperimentale dell’Abruzzo e del Molise (IZSAM).

The NDB contains data on all the holdings (herds, slaughterhouses, assembly centres, staging points, markets and pastures) and information relating to the animals and their movements from birth to death or slaughter, indicating the date of movement and the holdings of origin and destination.

Data on animal health were collected from the National Animal Health Managing Information System (SANAN) which is managed by IZSAM. SANAN stores all information about surveillance or eradication activities at national or regional level for those diseases for which a control/eradication program is in force (e.g. bovine tuberculosis, bovine brucellosis, enzootic bovine leucosis, schmallenberg disease, etc.). Data managed by SANAN refer to all activities and results thereof at farm and individual level.

Data considered in this study were the following:

1. Cattle movements recorded in NDB in 2009. Each movement record includes: origin and destination holdings (unique identifier of the holding, post address and geographic coordinates), type of holding (herd, slaughterhouse, market, staging point and pasture), date of movement, number of animals moved, number of animals present in the holding on the date of movement, number of females aged at least 18 months and present in the holding at the date of movement.
2. Brucellosis infected herds extracted from SANAN. In particular, data on brucellosis outbreaks occurred in Sicily in 2009 were used. All data recorded included: code of infected herd, date of infection, dates of herd inspection before and after the infection date, number of animals present (susceptible, tested, infected, culled), and dates of culling and slaughtering.

### Construction and characterization of the transmission network

The transmission network was generated using a brucellosis infected herd as seed and by extracting from the NDB all animal movements from the date of the most recent negative control and with a forward depth of 365 days. The sub-network includes the connections departing from the seed node and the connections among destination nodes until the fifth level of depth during the 365 days following the last negative control. Slaughterhouse nodes and foreign countries were removed because they are not contributing to the spread of the disease. The TN is a sub-network of 2009 Italian cattle trade network, already described by [11].

The transmission network analysed was designed considering holdings as nodes and animal movements between holdings as arcs.

**Fig 1:**
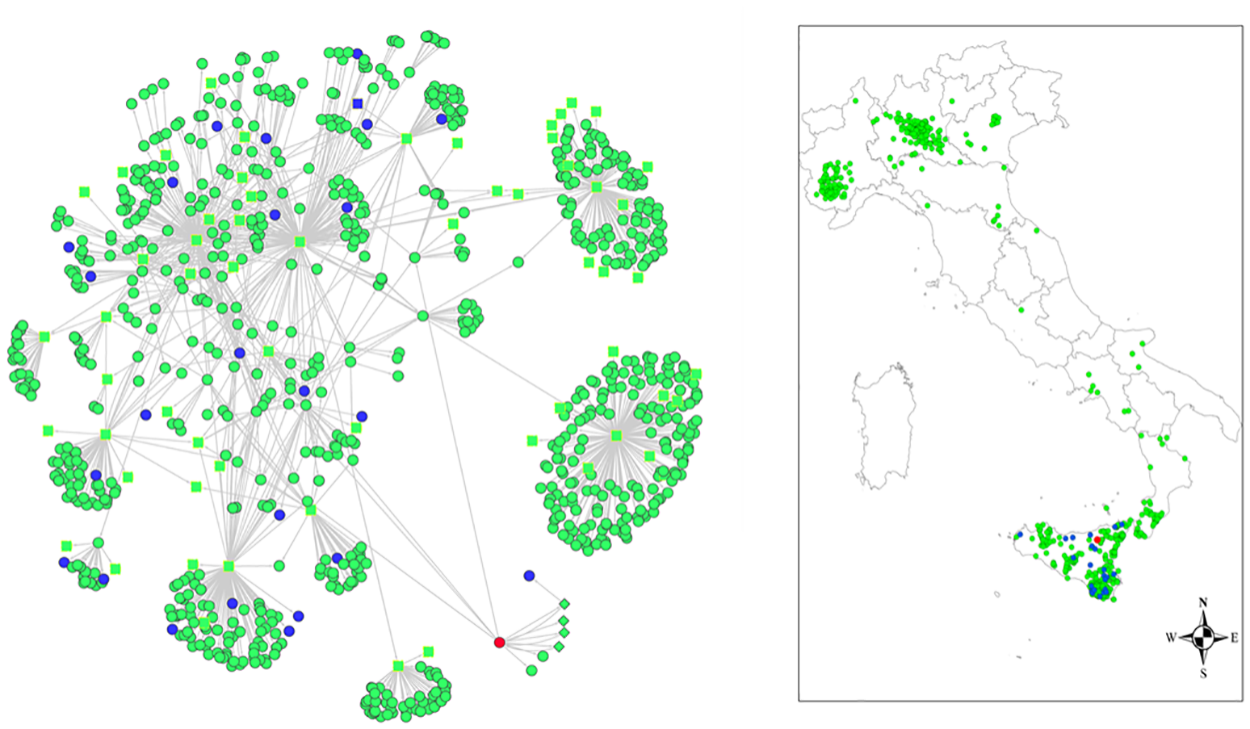
Graphic description of the static transmission network. The figure shows the graphic description of TN and the geographical node distribution. The holdings are nodes (green circles in the figure) and the movements of animals from holding to holding are arcs. The arc is oriented from the holding of origin to the holding of destination of the animal movement. The node shape characterizes the type of holding: diamonds represent pastures, squares represent markets and staging points, green dots represent herds. Moreover the blue dots represent the real brucellosis infected herds and the red dot represents the seed (the first brucellosis outbreak) detected in Sicily during 2009.

The static transmission network is composed by 899 nodes and 1195 arcs (the corresponding dynamic form consists of 2296 multiple arcs) and includes twenty six (considering the seed) brucellosis infected herds occurred in Sicily in 2009 (Fig 1).

Table 1 reports the number of nodes per type of classification in the TN and the percentage of animal moved and animal movements to each destination type.

**Table 1:** Number of nodes per type of holding in the transmission network and percentage of animal moved and animal movements to each destination type.

Type	No. of Nodes	% of animal moved to each type	% of animal movements to each type
Herds	832	10.6%	27.4%
Staging point and markets	54	89.3%	72.4%
Pastures	4	0.1%	0.2%

The sub-network was first analysed to investigate the structure (or topology) and to calculate the main centrality measures. Measures of cohesiveness at network-level (density, average path length and average clustering coefficient) were calculated and reported in Table 2. To characterize the TN at node level, the static centrality measures such as degree and betweenness measures were calculated. Moreover the hub and authority values were calculated respectively as described in Kleinberg [19].

**Table 2:** Network topological properties of the sub-network (TN).

Network property	Value
Density	0.0014
Characteristic path length	3.28
Average clustering Coefficient	0.031

The centrality measures have been used to optimize the veterinary control measures put in place in case of disease spread (e.g. block of animal movements from the market and staging points). The effects of removing the nodes with high values of centrality measures and their arcs from the network were evaluated through six scenarios arising from targeted node’s selection based on 98° percentile of hub, authority, degree, out-degree, in-degree and (cut-point) betweenness values.

The targeted selection approach is very useful in terms of prevention of the spread of an epidemic and its advantages have been widely described in the published literature [11–15].

In case of an epidemic, besides the fragmentation of the network, the timely identification of all secondary outbreaks and their extinction is fundamental to limit the damage to the epidemic itself and to limit the duration of the trade disruption due the fragmentation of the network.

### Implementation and performance evaluation of epidemiological models

Three different modelling approaches were evaluated in their capability of optimizing activities for the tracing of secondary outbreaks by the veterinary services, namely: the DFC model, a deterministic SIR model and a stochastic SEIR model.

### Disease flow centrality (DFC) on the empirical contact tracing network

DFC model is able to characterize in a more efficient way than the classical centrality measures the risk of disease spread across network and allows to simulate a simplified epidemic process. DFC identifies key holdings, in terms of risk and vulnerability (namely traversal and reachable DFC), in the dissemination of the infection taking into account for valid temporal paths. To identify nodes that are more likely to have been achieved by infection, in this work we used the reachable version of DFC [20].

### Deterministic SIR model on the empirical contact tracing network

The deterministic model is a network-based meta-population model having two different components: an intra-population (at holding level) and an inter-population (between holdings).

The intra-population model is a Susceptible-Infectious-Recovered (SIR) discrete-time model described by the following system of difference equations [11,21]:

$$\left\{ \begin{array}{l} S_{t+1}=S_{t}-\lambda \times S_{t}\\ I_{t+1}=I_{t}+\lambda \times S_{t}-rI_{t}\\ R_{t+1}=R_{t}+rI_{t} \end{array}\right.$$ (1)

where the time-step is 1 day, $\lambda =\;\beta \text{ x}\frac{\text{I}}{\text{N}}$ is the force of infection rate and β is the mean daily contact rate set to 0.027 [21,22]. If we assume that an infectious animal moving from node A to node B make node B infected, the inter-population component is described by the following equation:

$$I_{Bt+1}=m_{AB}\frac{I_{At}}{N_{A}}$$ (2)

where *I*_*Bt+1*_ is the number of infected animals entered in the node B at the time t+1, *N*_*A*_ is the population size of node *A* and *m*_*AB*_ is the number of moved animals from node *A* to node *B* at time *t*. The total node population is constant trough time, therefore after each movement the population is rescaled to the initial value.

### Stochastic SEIR model on the empirical contact tracing network

Modifications to the previous basic approach were made to take into account the variability in the infectious contacts between individuals (intra-population) and between holdings (inter-population). Moreover, an additional compartment of Exposed (E(t)) has been added, to identify infected animals not able to infect yet.

The Susceptible-Exposed-Infectious-Recovered (SEIR) stochastic discrete-time model (intra-population) is described by the following system of difference equations in which new animals in each compartment follow a binomial distribution:

$$\{\begin{array}{l} S_{t+1}=S_{t}-Binomial\left(S_{t},\lambda_{d}\right)\\ E_{t+1}=E_{t}+Binomial\left(S_{t},\lambda_{d}\right)-Binomial\left(E_{t},\alpha_{d}\right)\\ I_{t+1}=I_{t}+Binomial\left(E_{t},\alpha_{d}\right)-Binomial\left(I_{t},r\right)\\ R_{t+1}=R_{t}+Binomial\left(I_{t},r\right) \end{array}$$ (3)

Table 3 reports the epidemiological parameters used in the SEIR model. The probability of an animal becoming infected with brucellosis is not constant over time, as it is in many other diseases. Similarly, the duration of the incubation period is not constant over time. These time variations are due to the fact that the transmission of brucellosis occurs at the moment of parturition or abortion and the amount of brucellae eliminated by an infected animal decreases with the time elapsing from the moment of parturition or abortion.

**Table 3:** The epidemiological brucellosis parameters used in the stochastic SEIR model.

**β**	The monthly infectious contact rate is transformed in a daily contact rate. It is calculated starting from an abortion or birth event. The monthly infectious contact rate is: 0.95, 0.9, 0.8, 0.6, 0.5, 0.5, 0.3, 0.2, 0.1, 0.1, 0.1, 0.1 (months: 1 to 12)	[22, 23]
**α**	The monthly incubation rate (1/ α = the disease incubation period) is transformed in a daily incubation rate. It is calculated starting from an abortion or birth event. The monthly infectious contact rate is: 0, 0.001, 0.001, 0.1, 0.75, 0.8, 0.75, 0.5, 1 (months: 1 to 9)	[22, 23]
**r**	Recovery rate (1/r = the mean infective period). Brucellosis infected animals after puberty typically do not recover (r=0)	[24]

Therefore, the force of infection (λ_d_ = β_d_*[I/N]) depends on a contact parameter (β_d_), which on turn is variable (decreasing) from the moment when an infected animal delivers or aborts within the herd. The value of β by month after the parturition/abortion of the infected animal is shown in Table 3.

The elimination of brucellae by an infected animal (and, thus, the infectivity period) starts when an animal delivers or aborts. Therefore, the spread of brucellosis is closely related to the incidence of abortion, or parturition after infection. The occurrence of abortion depends on when the cow becomes infected. In general, abortion caused by brucellosis infection occurs between the fifth and eighth month of pregnancy. The probabilities of abortion in each month of pregnancy (α) were reported in Table 3.

For the sake of computational simplicity we did not implement an agent-based-model but for each time-step of 1 day, we extracted, randomly and with replacement, a sample of E_t_ and I_t_ size from vectors of daily values of βand αrespectively. Then the averages (β_d_, α_d_) of the sampled values were used. The aim of the type of model implemented is to take into account the increased variability due to the heterogeneity of the parameter values.

The daily values of β and α were calculated from their corresponding monthly values [22, 23] by the following formula:

$${\left(1-x_{d}\right)}^{30}=1-x_{m}$$ (4)

where *x*_*d*_ is the daily value and *x*_*m*_ is the monthly value.

**Fig 2:**
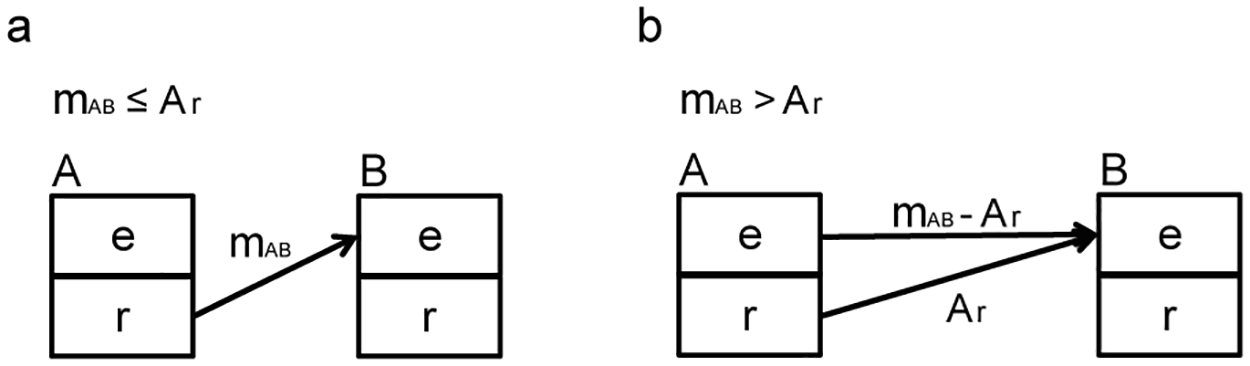
Graphic illustration of population subdivision role on moved animals. The node population consists of two sub-populations: the sub-population "r" of resident animals, and the sub-population "e" of animals introduced in the node. The panel ‘a’ represents the case of the number of moved animals from A to B is less than animals in “r” of A (m_AB_ ≤ Ar). Panel ‘b’ shows the case of the number of moved animals from A to B is more than animals in “r” of A (m_AB_ > Ar).

The node population has been divided into two sub-populations: the sub-population "r" consists of resident animals at the considered time-step and the sub-population "e" is constituted by animals introduced in the node at the same time-step. The choice to divide the population into two sub-populations was made to avoid moving animals that have just been acquired by the breeding and rearing nodes Fig 2a.

When the population "r" is smaller than the total number of animals to be moved, the missing animals are taken from the population "e". All moved animals increase the "e" population of the destination node Fig 2b.

In addition, for non-breeding or rearing nodes (markets, pastures and staging points), all the population belongs to the "r" group, including all received animals.

The dissemination of disease between nodes (inter-population component), was modelled after the intra-population spread by a multivariate hypergeometric distribution of animal movements:

$$\left(S_{AB},E_{AB},I_{AB},R_{AB}\right)=\;MultivariateHypergeo\left(m_{AB},\;S_{A},E_{A},I_{A},R_{A}\right)$$ (5)

where *m*_*AB*_ is the number of moved animals from node A to node B at each time-step.

Since the realistic time order of the movements within the daily network is not known, these are sorted in order to consider first all movements toward the staging points, collection centers, pastures and markets and, finally, the order of all the movements related to herds are chosen randomly.

At each time-step the demographic changes (births and deaths) are run on the basis of empirical data in the inter-population component.

We run the stochastic model for 7.500 iterations, each simulating one-year time frame.

### Comparison of performances of the approaches considered

To compare the ability of the models to identify outbreaks and healthy farms correctly, we used the following results from simulations:

We recorded the number of infected animals per node at final time (nI) for the SIR model; the node state, i.e., if a node has at least one exposed or infected animal in the one-year frame (HI), and the maximum of the exposed plus infected animals per node (mEI) for the SEIR model.

We then transformed these results in the indicators shown in Table 4.

**Table 4:** Model results and ROC variables.

Model	Recorded results	ROC variables
**SIR**	nI	(a): nI
**SIR**	nI	(b): nI/node population
**SEIR**	HI	(a): sum(HI)/7500
**SEIR**	mEI	(b): average(mEI)/node population

We performed the comparison of the epidemiological models and DFC to prioritise the intervention on the farms of the network by using their ROC curves and calculating the values of the AUC and confidence limits (95%) [25].

In the case of brucellosis outbreak, veterinary services check for infection in farms within a distance of three kilometres from the primary outbreak and in farms that had received animals from it. They repeat the procedure if secondary outbreaks are detected in the checked farms (Ireneo Sferrazza, head of the veterinary services of the province of Enna, personal communication). We used this criterion and evaluated its sensitivity and specificity. To compare correctly ROC curves and on-field veterinary activity, results are focused on Sicily region farms, being veterinary activity is different between Italian regions.

The statistical R software [26] and package ‘igraph’ v. 1.0.1 [27] was used to perform the network analysis and to implement the algorithms to calculate hub and authority centralities. The network was visualized using Cytoscape software [28,29].

Simulation models were implemented and analysed using R software [26] and EpiTrace [15,29].

## Results

### The targeted node’s selection scenarios applied to the transmission network

**Fig 3:**
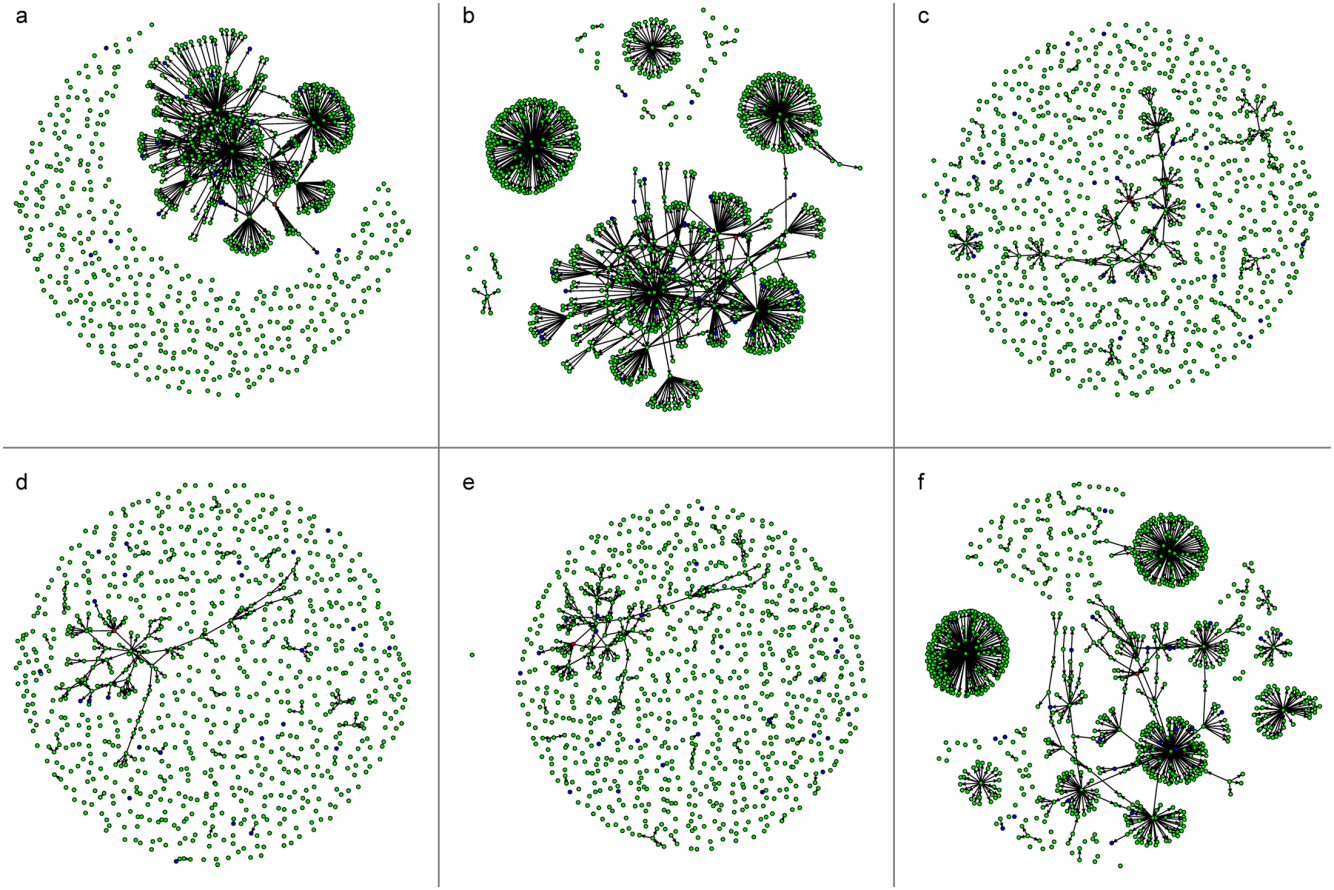
Results of the six scenarios proposed in terms of TN reduction. Fragmented networks resulting from the blocking the movements of the holdings with the highest values of the centrality measures (≥ 98° percentile): a. authority, b. hub, c. cut-point, d. degree, e. out-degree and f. in-degree.

To characterise the TN at node level the static centrality measures: hub, authority, (cut-point) betweenness, degree, out-degree and in-degree values were calculated. The effects of removing the nodes with high values of centrality measures (> = 98° percentile) and their arcs from the network were estimated through six scenarios to prevent possible disease spread and the resulting network fragmentation is shown in Fig 3.

To characterise the TN at node level the static centrality measures: hub, authority, (cut-point) betweenness, degree, out-degree and in-degree values were calculated. The effects of removing the nodes with high values of centrality measures (> = 98° percentile) and their arcs from the network were estimated through six scenarios to prevent possible disease spread and the resulting network fragmentation is shown in Fig 3.

The six scenarios considered were: a. removal of the hub, b. authority, c. (cut-point) betweenness, d. degree, e. out-degree and f. in-degree and their results as the percentage of TN reduction and relate reached outbreaks are reported in Table 5.

**Table 5:** The six scenarios centrality measures based results.

Scenarios	TN reduction	Reached outbreaks
**a.**	45%	84%
**b.**	16%	84%
**c.**	80%	12%
**d.**	85%	8%
**e.**	84%	8%
**f.**	37%	4%

The in-degree scenario shows a minor network reduction of 37% (that expresses the smallest damage for trade among the six scenarios) and the lowest percentage of reached outbreaks (4%) post network fragmentation. It is, therefore, the best approach to prevent possible disease spread in the network TN.

### Evaluation of the epidemiological model performances

**Fig 4:**
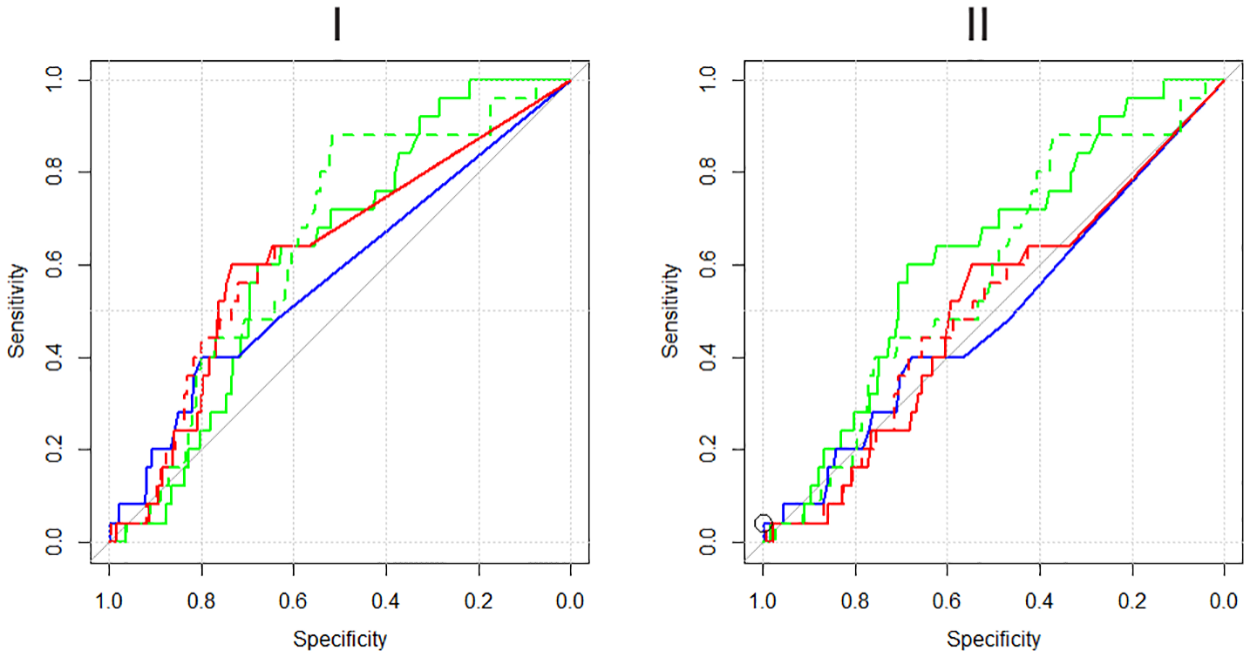
ROC curves. ROC curves for the methods considered to prioritise the field activities of outbreak tracing. DFC (blue), SIRa (green), SIRb (dashed green), SEIRa (red) and SEIRb (dashed red). Differences in performances when using the entire TN (panel I) and the TN of Sicily only (panel II) are shown. The black dot represents the Sensitivity and Specificity of the veterinary services approach.

The comparison of the epidemiological models and DFC approach to prioritise the intervention on farms for early outbreak detection was performed using ROC curves (Fig 4) and calculating the values of the AUC and confidence limits (95%) (Table 6). We used the variables reported in Table 4 for the SIR and SEIR models ROC calculation.

**Table 6:** The AUC values for the entire TN and that of Sicily only, and the confidence limits (95%) in brackets.

Model	AUC (TN)	AUC (Sicily)
**DFC**	0,58 (0,46–0,7)	0,49 (0,37–0,61)
**SIRa**	0,63 (0,54–0,71)	0,62 (0,52–0,72)
**SIRb**	0,65 (0,55–0,74)	0,58 (0,47–0,68)
**SEIRa**	0,62 (0,52–0,73)	0,5 (0,39–0,61)
**SEIRb**	0,63 (0,52–0,73)	0,5 (0,39–0,62)

The figure shows the ROC curves calculated using the model results on the entire TN (Fig 4I), and on the Sicily region only (Fig 4II), respectively. Also, the AUC calculation follows the same criteria.

The need to test the model performances in Sicily comes from the fact that the brucellosis is not present in the north of Italy, the animal husbandry in northern Italy is different from that in Sicily, and the seed did not send infection to northern Italy. The negative nodes in the territories of the north may affect the test results. Moreover, the regional analysis allowed to correctly compare ROC curves with the field activity of the Sicilian veterinary services.

The SIR model performed better than the other models, both in the entire TN (SIRb, panel I) and in the TN of Sicily only (SIRa, panel II), whereas the DFC showed the worst performance.

The models' efficiency when evaluated using the AUC was poor for all models. However, their performance was slightly better when we consider the entire TN than the TN of Sicily only (Table 5).

The lower confidence limit of DFC included the 0.5 value, while the lower limits of all other models are slightly greater than 0.5 for the entire TN. When considering the TN of Sicily only, the models’ performance becomes further worse: all models, except the SIRa, showed a lower confidence limit that includes 0.5.

The criterion adopted by the field veterinary services resulted in sensitivity and specificity values of 4% and 99,8% respectively. These values did not improve when the radius of the buffer doubled from the 3 km adopted by the veterinary services to 6 km. Farms within a distance of 3 and 6 kilometres from the seed are 30 and 100 respectively. These results are affected by the method and the number of farms to be checked under financial and time constraints. Indeed, on average, the veterinary services can monitor from six to ten farms per month.

For this reason, we compared the number of secondary outbreaks identified by the different approaches per fixed number of controlled farms. When the number of farms is limited to 30, all the methods identify only one secondary outbreak. Therefore, the minuscule number of 30 farms to be controlled does not highlight the differences in the ROC curves and leads to a useless model comparison criterion. On the contrary, the use of 100 farms (corresponding to a 6 km radius of the veterinary services buffer), allowed a more efficient discrimination of the methods.

**Fig 5:**
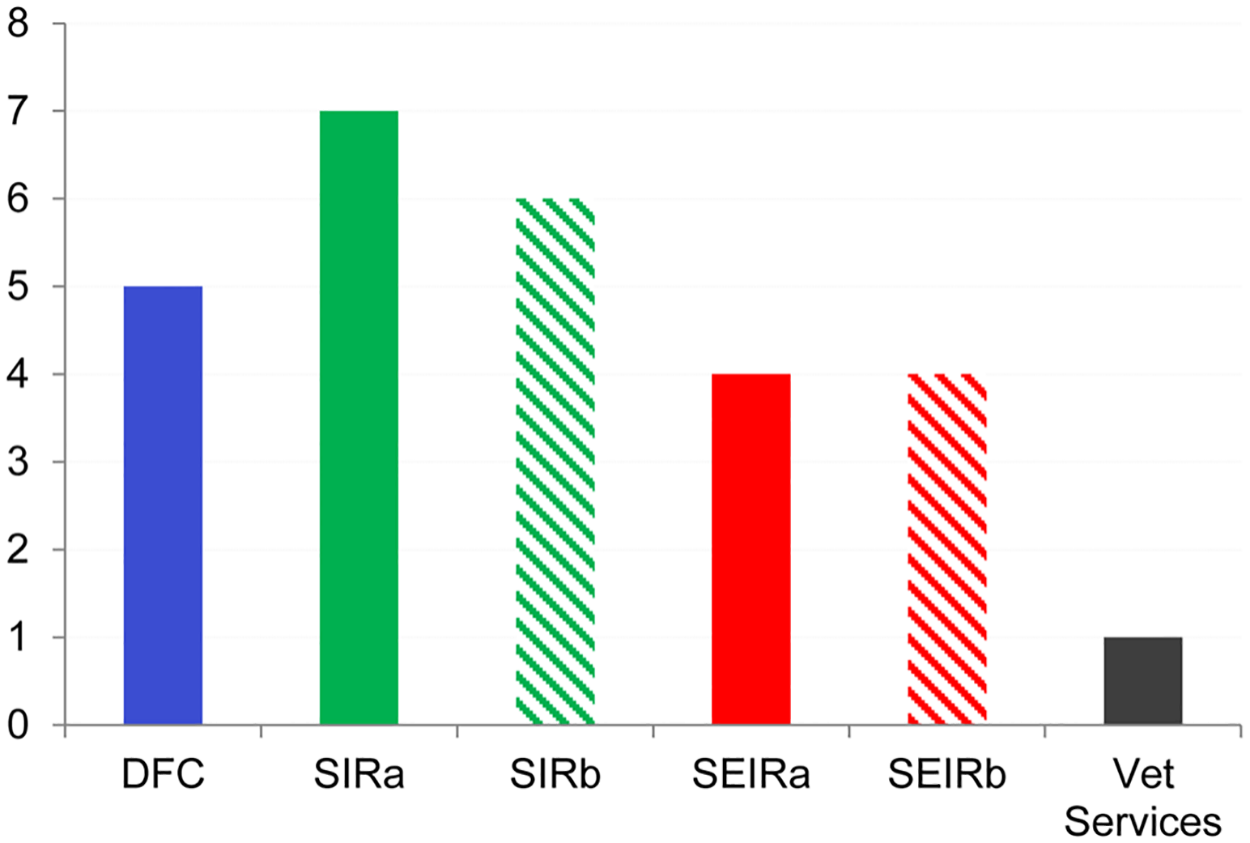
Outbreaks detected for a set of 100 checked farms. Number of outbreaks detected by each of the models (DFC, SIR and SEIR) on a set of 100 checked farms. SIR model is the most efficient detecting 7 secondary outbreaks, against the only one identified by veterinary services method.

SIR model is clearly the most efficient (Fig 5) detecting 7 secondary outbreaks, against the only one identified by veterinary services method.

In summary, the criterion followed by the field veterinary services lacks sensitivity and is inefficient as concerns cost-benefit ratio. All methods proposed by the authors to define the priorities of intervention are more effective than that now followed in the field. The most efficient among the set of proposed methods is the SIR, which gives the highest sensitivity, still requiring the lowest computation effort.

## Discussion

Data about animal movements and the resulting contacts between holdings can be represented in the form of networks, and several studies in the veterinary sector have demonstrated how the spread of diseases is influenced by the nature and structure of these contact networks.

The application of NA and network tools, such as the classification of nodes based on a set of centrality measures, may be useful for planning prevention activities. These prevention activities are aimed at fragmenting the network and decreasing the number of reachable nodes by potentially infected animals. These prevention activities, however, have a series of limitations, namely:

- The initial fragmentation of the network determines a disruption of the trade in the affected area and cannot be strictly enforced for long time;
- There is a spontaneous restructuring of the network itself through changes of the behaviour of farmers that makes the effect of the network fragmentation transient;
- More important, in many cases, the detection of the infection in a farm occurs long time after the entry of the infection in the farm itself. In such cases, most of the spread has already occurred when the outbreak is detected. This occurs with infections characterised by absent/mild clinical signs and regulated by control or eradication programs based on the periodic (annual) testing of farms, such as the bovine brucellosis. When the infection of a farm is detected, it could have originated any time during the past year since the last control. In this context, the use of prevention measures based on the block of nodes with high values of the centrality measures is of limited or no value in the control of disease.

Therefore, in all these cases it is necessary to put in place concurrent actions to identify and eradicate sources of the infection already present in the trade network. These actions will allow to limit the period during which it is necessary to enforce the movement restrictions.

In our field conditions, the concurrent actions put in place by the veterinary services are based on the control of the neighbouring farms and first order contacts. No attempt is usually made to follow the contact network beyond the first order.

Our study was aimed at assessing the efficiency of three different approaches in the prioritization of controls of farms in-contact with an infected one. All approaches started from the identification of the entire sub-network originated from an infected farm, up to the fifth order of contacts. In our case, the sub-network (up to the fifth order of contacts) represents approximately the 25% of the entire Sicilian network. The decision to limit the sub-network TN to the fifth order of contacts depended on computational needs (the execution time of one iteration of stochastic SEIR model was on average of two minutes).

Three different epidemiological models were applied to estimate the probability of infection of each farm of the sub-network. These models were: the DFC model based only on temporal cattle network structure, a deterministic SIR model and a stochastic SEIR model (including the intra- and inter- population(s) brucellosis spread).

Our sub-network consists of 25 secondary outbreaks, none with a direct link with the seed. One secondary outbreak has a geodesic distance of two from the seed; eight have a distance of three; sixteen have four links from the seed. Most of the secondary outbreaks pass through trade stables that did not experience brucellosis. The only outbreak detected by the criterion used by the veterinary services was identified for its geographic proximity to the seed.

Despite the ability to detect all secondary outbreaks with each of the approaches considered, the efficiency in the their identification as high priority farms was generally poor, and variable with the various methods.

**Fig 6:**
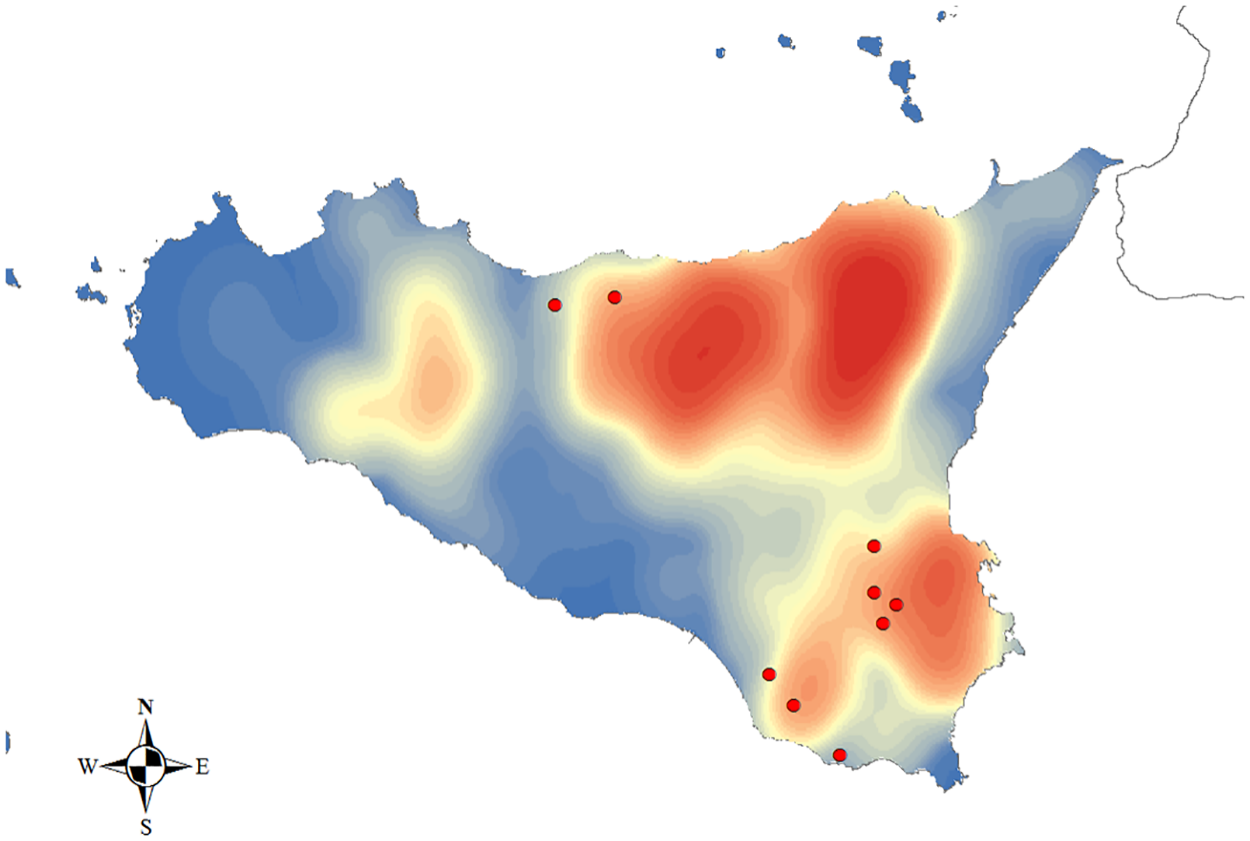
Pasture density map and outbreaks not detected by SEIR model. All outbreaks not detected by the SEIR model (9 on 25) fall in the high density zones of pastures of Sicily region.

We can say that the efficiency was generally poor because the AUCs of the methods were between 0.58 and 0.65 when we consider the entire TN, and between 0.49 and 0.62 when we consider the TN of Sicily only. This reduced efficiency has the consequence that a great number of the farms belonging to the sub-network need to be checked to detect all secondary outbreaks. These results is not surprising and may be related to several factors:

- Pasture contacts play a major role in the transmission of the disease [6] that cannot be revealed by the models. This phenomenon occurs exceptionally in Sicily because, despite the presence of many pastures, the respective movements are missing or only partially registered in the NDB. Fig 6 shows the pasture density map and outbreaks that the SEIR model was unable to detect in Sicily;
- Cases of illegal movements cannot be excluded, although in recent years systems of traceability for cattle in NDB have reached a good level of reliability [20];
- Indirect contacts between farms through fomites (exchange of materials or personnel, etc.);
- Husbandry practices, farming system, lack of farm biosecurity and sanitation, temporary exchange of animals for mating reasons could contribute to inter-herd brucellosis spread;
- Limitation of the network to the fifth order excludes some possibly infectious paths between outbreaks.

Anyway, the AUCs are significant higher than 0.5 and indicate that the considered methods improve the ability of detection of the infected farms.

Differences among ROC curves reflect the different nature of the three proposed approaches.

DFC is the simplest model because it relies only on the network of animal movements and it does not include specific parameters for the disease under study; therefore DFC represents the system under study in the least realistic way. For this reason, it was expected to have the lowest performance values.

Deterministic SIR model is not limited to the use of movement data, but also considers the epidemiological disease parameters. It was also the most sensitive approach because every movement involving an infected source, spread the disease to the farm of destination. The deterministic SIR model has the advantage of having the least computation requirements and the shortest time.

Stochastic SEIR is the most complex model, and it represents the disease spread in the most realistic way. In addition to the compartments considered by the SIR model, the SEIR has an additional compartment comprising the infected animals not able to infect yet. Moreover, the type of SEIR model that we implemented was stochastic, so considering also the intra- and inter- population(s) uncertainty and traders’ behaviour. Despite its greater realism compared to the two other models, its ability in detecting secondary outbreaks, did not improve, as shown by ROC curve. The low sensitivity is probably due to the increased time elapsed from susceptible to infected (and infectious) state, through the exposed compartment. It still retains a high specificity, even greater than the SIR model. The stochastic SEIR model simulates the animal movements on a discrete scale, while the deterministic SIR model, uses a continuous scale. The movement of discrete animals further decreases the chances of moving the infection between herds. In the comparison between the entire TN and the TN of Sicily only, the SEIR model shows a higher specificity in the case of the entire TN model due to the lower number of negative nodes located in northern Italy.

In conclusion, the use of the proposed methods improves the efficacy and efficiency of the tracing activities in comparison to the procedure currently adopted by the veterinary services. Our study provides the competent authority with the information required for the choice of the method to adopt, based on models’ performance and computation time. An overall assessment shows that the SIR model is the most suitable for the practical needs of the veterinary services, being the one with the highest sensitivity and the shortest computation time.
